# Effective Early Detection of Epileptic Seizures through EEG Signals Using Classification Algorithms Based on t-Distributed Stochastic Neighbor Embedding and K-Means

**DOI:** 10.3390/diagnostics13111957

**Published:** 2023-06-03

**Authors:** Khaled M. Alalayah, Ebrahim Mohammed Senan, Hany F. Atlam, Ibrahim Abdulrab Ahmed, Hamzeh Salameh Ahmad Shatnawi

**Affiliations:** 1 Department of Computer Science, College of Science and Arts, Najran University, Sharurah 68341, Saudi Arabia; 2 Department of Artificial Intelligence, Faculty of Computer Science and Information Technology, Alrazi University, Sana’a P.O. Box 1152, Yemen; 3 Cyber Security Centre, WMG, University of Warwick, Coventry CV4 7AL, UK; hany.atlam@warwick.ac.uk; 4 Computer Department, Applied College, Najran University, Najran 66462, Saudi Arabia; iaalqubati@nu.edu.sa (I.A.A.);

**Keywords:** EEG, epileptic seizure, DWT, K-means, PCA, t-SNE, machine learning

## Abstract

Epilepsy is a neurological disorder in the activity of brain cells that leads to seizures. An electroencephalogram (EEG) can detect seizures as it contains physiological information of the neural activity of the brain. However, visual examination of EEG by experts is time consuming, and their diagnoses may even contradict each other. Thus, an automated computer-aided diagnosis for EEG diagnostics is necessary. Therefore, this paper proposes an effective approach for the early detection of epilepsy. The proposed approach involves the extraction of important features and classification. First, signal components are decomposed to extract the features via the discrete wavelet transform (DWT) method. Principal component analysis (PCA) and the t-distributed stochastic neighbor embedding (t-SNE) algorithm were applied to reduce the dimensions and focus on the most important features. Subsequently, K-means clustering + PCA and K-means clustering + t-SNE were used to divide the dataset into subgroups to reduce the dimensions and focus on the most important representative features of epilepsy. The features extracted from these steps were fed to extreme gradient boosting, K-nearest neighbors (K-NN), decision tree (DT), random forest (RF) and multilayer perceptron (MLP) classifiers. The experimental results demonstrated that the proposed approach provides superior results to those of existing studies. During the testing phase, the RF classifier with DWT and PCA achieved an accuracy of 97.96%, precision of 99.1%, recall of 94.41% and F1 score of 97.41%. Moreover, the RF classifier with DWT and t-SNE attained an accuracy of 98.09%, precision of 99.1%, recall of 93.9% and F1 score of 96.21%. In comparison, the MLP classifier with PCA + K-means reached an accuracy of 98.98%, precision of 99.16%, recall of 95.69% and F1 score of 97.4%.

## 1. Introduction

Epilepsy is one of the most common neurological disorders worldwide. According to the World Health Organization, approximately 50 million people worldwide suffer from this disorder [1]. Epilepsy occurs due to brain neurons’ abnormal and sudden secretions [2]. Epilepsy is typically not a direct cause of death in most cases. However, it is important to note that epilepsy can increase the risk of certain potentially life-threatening complications. These complications include sudden unexpected death in epilepsy (SUDEP); SUDEP is a rare but significant risk associated with epilepsy. It refers to cases where a person with epilepsy dies suddenly and unexpectedly, and no clear cause of death is identified during autopsy. The exact mechanisms of SUDEP are not fully understood, but it is believed to be related to a combination of factors, including seizures affecting the respiratory or cardiovascular systems. They also include accidents and injuries; seizures can cause loss of consciousness, convulsions, or altered awareness, which can lead to accidents and injuries. Another complication is status epilepticus: a prolonged seizure or a series of seizures where the person does not regain consciousness between seizures. Status epilepticus is a medical emergency and requires immediate treatment. If not treated promptly, it can lead to severe brain damage or even death. It is worth mentioning that the overall mortality rate for epilepsy is generally low, and most people with epilepsy can lead full and productive lives with appropriate management and treatment. However, it is crucial for individuals with epilepsy to work closely with healthcare professionals to minimize the risks associated with the condition and manage it effectively [3]. The inability of brain neurons to regulate electric signals in the brain results in seizures, a condition that has been of interest to many researchers because of its complexity and seriousness. Seizures are usually accompanied by disorders in sensation, movement or mental functions [4]. Over 30% of patients with epilepsy still suffer from uncontrolled seizures despite treatment with antiepileptic drugs. Based on the areas of the brain that are activated during seizures, seizures are classified into two types: partial and generalized. A partial seizure originates from one region of the brain and remains in one hemisphere, whereas a generalized seizure affects the whole brain [5]. The electrical activity of the brain is represented by EEG signal waves originating from the brain’s neurons. EEG signal waves are recorded by placing non-invasive electrodes on the scalp. These signals display information on mental defects and neurological conditions [6]. The brain’s electrical signals can be analyzed through the five frequency bands produced by the EEG [7]: delta, alpha, theta, gamma and beta. Excessive discharge of electrical signals in brain cells produces abnormal seizures, which are one of the signs of epilepsy. Radiologists distinguish EEG signals caused by seizures from other factors via special discriminatory patterns, such as high-amplitude repetitive activities with a combination of slow and spike waves [8]. Hence, discovering these features is a difficult task, and following each EEG signal is troublesome and time consuming [9]. Therefore, an automated approach for detecting seizures to diagnose epilepsy in a timely manner needs to be developed [10]. EEG is an effective tool for detecting areas of differences in neuronal activity correlated with epilepsy. Accordingly, seizures can be detected by analyzing EEG signals. The key point in analyzing EEG signals is to extract the most important and most effective features that represent the characteristics of the signal [11]. Given that the characteristics of EEG signals have not been established yet, several methods for identifying EEG on the basis of time–frequency (TF) algorithms have been proposed for classification [12]. The present study established TF algorithms as one of the most important algorithms for discovering the behavior of different signals with different frequencies and times [13]. In particular, the DWT algorithm was applied to analyze nonstationary signals, such as EEG, to extract the most effective features. The PCA and t-SNE algorithms were employed to represent high-dimensional data in a low-dimensional spaces. Two models were built by applying the K-means clustering algorithm and PCA (K-means + PCA) and then with t-SNE (K-means + t-SNE). The important features were fed into five types of classification algorithms, which trained the dataset and tested the performance of the classifiers on a part of the dataset.

The main contributions of this paper can be summarized as follows:

- Features are extracted by decomposing signal components via the DWT algorithm and by applying PCA and the t-SNE algorithm to reduce data dimensions to obtain a feature vector in two ways, namely, DWT + PCA and DWT + t-SNE.
- The data set is divided into subgroups by using K-means clustering, with each group containing similar points. PCA and the t-SNE algorithm are then applied to reduce data dimensions to obtain feature vectors in two ways, namely, K-means + PCA and K-means + t-SNE.
- The hyperparameters of all classifiers have are adjusted to attain the best results in diagnosing epilepsy.
- These highly efficient algorithms are generalized to help physicians obtain a highly accurate diagnosis while saving time and effort.

The rest of the paper is ordered as follows: Section 2 discusses techniques used in the relevant literature. Section 3 describes the materials and methods with subsections for pre-processing data, feature extraction, and methods for reducing data dimensions. Section 4 provides and discusses the experimental results. Finally, the conclusions are provided in Section 5.

## 2. Proposed Approach for Detection of Epileptic Seizures

**Figure 1:**
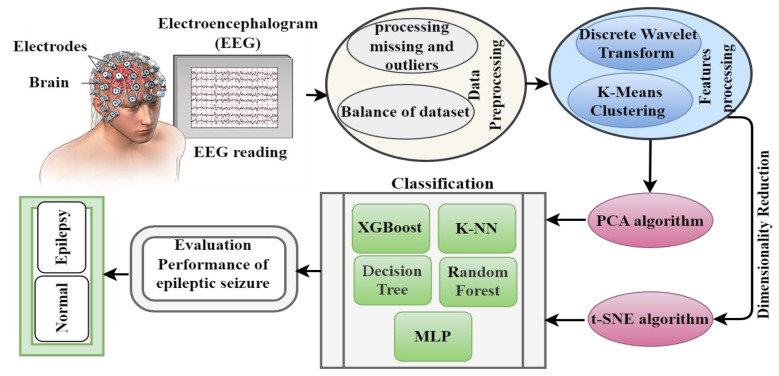
Methodology for analyzing EEG signals for early diagnosis of epileptic seizures.

Epilepsy is a common neurological disease that occurs due to the inability of brain neurons to regulate electrical signals in the brain, which leads to seizures. EEG is usually measured and evaluated by neurologists. Therefore, in this study, automated systems were developed to track EEG signals accurately to detect epileptic seizures efficiently. Figure 1 describes the methodology for diagnosing the epileptic seizure dataset. The dataset included all stages, from dataset acquisition, data cleaning, feature extraction, and signal component decomposition via the DWT method. The dataset was divided into subgroups, each containing similar data using K-means. The high-dimensional data was reduced and then put in low-dimensional spaces via PCA and the t-SNE algorithm. Finally, the low-dimensional dataset was classified using five classification algorithms: extreme XGBoost, KNN, DT, RF and MLP classifiers.

### 2.1. EEG Dataset

The epileptic seizure dataset used in this work was obtained from the machine learning repository UCI [14].

The Epileptic Seizure Recognition Data Set is a collection of EEG recordings used to recognize epileptic seizures. The dataset is available on the UCI Machine Learning Repository and consists of a single file named “data.csv”. The dataset consists of 5 folders, each representing a different patient. Each folder contains 100 files, with each file representing a recording of the brain activity of a single person. Each recording lasts for 23.6 s and contains 4097 data points. Each data point is recorded at a different point in time.

Each data point in a recording represents the electrical activity at a specific time. There are 178 numerical attributes for each data point. The attributes are obtained from 23.6 s of EEG (electroencephalogram) signals. The first attribute represents the patient ID, the second attribute represents the class label (0 for non-seizure activity; 1 for seizure activity), and the remaining 178 attributes represent the EEG signal data. Each piece of information contained 178 data points that represented the columns. The last column represented label y, which contained five classes. Each class contained 2300 data points (i.e., multiple classification). Each class was classified as a binary classification: class 1 represented epilepsy cases, whereas class 0 represented normal cases (a combination of four classes (1, 2, 3 and 4)) [15]. Thus, the dataset be-came unbalanced as epilepsy cases represented 2300 data points, whereas normal cases represented 9200 data points [15].

In this study, 80% of the dataset was allocated to training the systems and 20% to testing the performance of the systems. In a patient-independent seizure detection setting, the goal is to develop a seizure detection algorithm that can be generalized well to new, unseen patients. To achieve this, it is essential to ensure that the records or samples of the same patient do not appear in both the training and testing subsets. The reason for this separation is to evaluate the algorithm’s performance on unseen data accurately. If the same patient’s data were present in both the training and testing sets, it would introduce bias and artificially inflate the algorithm’s performance metrics. The model could potentially memorize specific patterns or characteristics of the patient’s seizures instead of learning generalizable features. By strictly separating the patient data into training and testing subsets, researchers can assess the algorithm’s ability to detect seizures in unseen patients, which is crucial for patient-independent seizure detection.

### 2.2. Data Preprocessing

Preprocessing is an essential step in data analysis and machine learning tasks. It involves transforming raw data into a format that is suitable for further analysis or modeling. The goal of preprocessing is to improve data quality, reduce noise, handle missing values, and extract relevant features from the raw data. It typically includes several steps, which can vary depending on the specific task and the nature of the data. Overall, the preprocessing steps are designed to enhance the quality, reliability, and usefulness of the data for subsequent analysis or modeling tasks. They play a crucial role in ensuring accurate and meaningful results in various data-driven applications [16].

#### 2.2.1. Missing Data

The epilepsy dataset did not contain lost data; thus, methods for replacing missing data were not applied.

#### 2.2.2. Balance of Dataset

The dataset was unbalanced as it contained 2300 data points (20%) for class 1 (epilepsy cases), whereas class 0 (normal cases) contained 9200 data points (80%). Therefore, the classes were balanced to obtain proper diagnostic accuracy. In this study, the classes of the dataset were balanced via the oversampling technique.

Oversampling is a technique used to balance an imbalanced dataset by increasing the number of instances in the minority class. It involves replicating or creating new synthetic samples from the existing minority class samples until a more balanced distribution is achieved. The goal is to provide the machine learning model with sufficient examples of the minority class to improve its performance [17].

The following is a general overview of how oversampling works:

- Identify the minority class: In an imbalanced dataset, there is a class imbalance, meaning one class has significantly fewer instances than the other class(es). The minority class refers to the class with fewer instances.
- Replicate samples: The existing samples from the minority class are replicated or duplicated to increase their representation in the dataset. This is achieved by randomly selecting instances from the minority class and adding them as additional samples.
- Synthetic sample generation: The approach is to generate synthetic samples using techniques such as the synthetic minority oversampling technique (SMOTE). SMOTE creates new samples by interpolating between existing minority class samples. It selects a minority class instance, identifies its k-nearest neighbors, and generates synthetic samples along the line segments connecting the instance with its neighbors.
- Repeat until balance is achieved: The replication or synthetic sample generation procedure is repeated until the preferred balance between the minority and majority class is achieved. This can be determined based on a specific ratio or until a satisfactory balance is obtained.
- Model training: The oversampled dataset, now with a more balanced distribution, can be used to train a machine learning model. The model can learn from the increased number of minority class examples and make better predictions on new, unseen data.

It is important to note that oversampling techniques should be used with caution and evaluated carefully. Oversampling can potentially introduce bias or overfitting if not applied properly. It is recommended to combine oversampling with other techniques such as cross-validation, regularization, or undersampling the majority class to further improve the performance and robustness of the model.

After applying this technique, the model obtained 18,400 records for the two classes divided into 9200 records for class 1 (epilepsy cases) and 9200 records for class 0 (normal cases). Thus, the epilepsy dataset became balanced.

### 2.3. Exploratory Data Analysis

Apart from finding the mean, standard deviation and maximum and minimum values, exploratory data analysis is a statistical method that can be used to analyze a dataset and summarize the most important features and determine the correlation rate between these features [18]. The dataset contained 11,500 data points (rows) and 178 features (columns). Table 1 describes the statistical measures of the epilepsy dataset.

#### 2.3.1. Correlation Features

Statistics is one of the methods used in data processing to interpret raw data and make them understandable. Using descriptive statistics, data can be represented in the form of tables or graphs that are easy to understand. In this study, the correlation between the features recorded by EEG signals was determined, and the correlation between each EEG data point and the other was extracted [19]. Moreover, each data point’s correlation with the target’s feature was identified.

Correlations between features in a CSV dataset indicate the statistical relationship or association between different attributes or variables. They measure how changes in one variable relate to changes in another variable. Correlation is typically measured using a correlation coefficient, such as Pearson’s correlation coefficient.

The strength of correlation between features can be categorized as follows:

**Strong Positive Correlation:**

When two features have a strong positive correlation, it means that as one feature increases, the other feature also tends to increase proportionally. The correlation coefficient will be close to +1, indicating a strong positive relationship.

**Weak Positive Correlation:**

A weak positive correlation means that there is a positive relationship between features, but it is not as strong as in the previous case.

The correlation coefficient will be between 0 and +1, closer to 0 than to +1. It indicates that as one feature increases, the other feature tends to increase, but not as consistently or strongly.

**No Correlation:**

When there is no correlation between features, it means that changes in one feature do not have a consistent relationship or impact on the other feature. The correlation coefficient will be close to 0, indicating no significant relationship.

**Weak Negative Correlation:**

A weak negative correlation implies that as one feature increases, the other feature tends to decrease, but the relationship is not strong. The correlation coefficient will be between 0 and −1, closer to 0 than to −1.

**Strong Negative Correlation:**

When two features have a strong negative correlation, it means that as one feature increases, the other feature tends to decrease significantly. The correlation coefficient will be close to −1, indicating a strong negative relationship.

When calculating the correlation between features (independent variables) and a classification (dependent variable), using measures of association specifically designed for data is common.

These measures of association help determine the relationship between the features and the classification in terms of their categorical nature. They provide insights into how closely related the features are to the classification outcome. However, it is worth noting that correlation measures for categorical data are not as commonly used as they are in the case of continuous variables.

#### 2.3.2. Discrete Wavelet Transform 

The DWT is a signal processing technique that decomposes a signal into different frequency bands, providing both time and frequency information. DWT has been used in the context of seizure detection due to several justifications and potential benefits it offers.

Multiresolution analysis: DWT allows the decomposition of a signal into different scales or resolutions, which can capture both high-frequency details and low-frequency trends. Seizure activity often manifests as transient high-frequency components superimposed on low-frequency trends. By decomposing the signal with DWT, it becomes possible to analyze these components separately, potentially enhancing the detection of seizure-related patterns.

Time-frequency localization: DWT provides good time and frequency localization compared to other frequency analysis techniques. Seizure events can occur over a range of frequencies and durations, and DWT’s ability to localize these events in both time and frequency domains makes it a suitable tool for identifying seizure-related features.

Feature extraction: DWT can extract features from different frequency sub-bands that may contain discriminative information for seizure detection. These features can include energy distribution, wavelet coefficients, or statistical measures computed from the decomposed sub-bands. By extracting relevant features from the DWT coefficients, it becomes possible to characterize seizure activity and distinguish it from non-seizure patterns.

Noise reduction: DWT can help denoise the signal by separating noise components from the underlying signal. Seizure detection systems often face challenges related to artifacts and noise, which can interfere with accurate detection. By applying denoising techniques based on DWT, it is possible to reduce the impact of noise on seizure detection algorithms and improve their performance.

Regarding performance, the effectiveness of DWT in seizure detection depends on several factors, including the specific implementation, feature extraction methods, and the classification algorithm used in combination with DWT. While DWT provides a powerful tool for signal analysis, its performance may vary depending on the dataset characteristics and the complexity of seizure patterns.

EEG have many nonstationary or transitory properties. Thus, frequency and time methods, such as the wavelet transform method, must be used. Various functions are used to analyze waves as in Equation (1).

(1) $$W\left(a,b\right)=\int_{-\infty }^{\infty }x\left(t\right)\frac{1}{\sqrt{a}}\psi (\frac{t-b}{a})$$

where a changes the time scales of the probing function, and b translates the function through *x*(t), *ψ.* If a is greater than 1, then the wavelet function $W\left(a,b\right)$ stretches along the time axis; if it is between 1 and 0, then it makes contact with the function. The probing function *(ψ*) can be any of the different functions, and it takes an oscillating form, which is why it is called a “wave”. The DWT method constrains the contrast in scale and translation with powers of 2. The DWT algorithm decomposes the signals through filter banks. The filter group is used to split the signal into different spectral components, a procedure called sub-band coding. Each stage contains two down-samplers and two filters. D1 details are taken from a high-pass filter and A1 approximation is carried out via the low-pass filter. Choosing the appropriate wave and number of signal decomposition levels is vital in signal analysis via the DWT method. Frequency components determine the number of levels and the selection of levels so that the parts of the signal required to classify the signal are preserved. Given that EEG signals do not have components of frequency above 30 Hz, their number of levels is 5. Therefore, EEG signals are decomposed into D1–D5 details and A5 approximation [20].

The DWT has several decomposition levels, resulting in approximation (A) and detail (D) coefficients at each level.

Below is an explanation of the terms commonly used in DWT:

A1 (Approximation 1):

A1 represents the approximation coefficients at the first level of the DWT decomposition.

It captures the signal’s low-frequency components and approximates the original signal at a coarser resolution.

A1 corresponds to the low-pass-filtered version of the input signal.

D1-D5 (Detail 1 to Detail 5):

D1 to D5 represent the detail coefficients at different levels of the DWT decomposition.

Each level of decomposition captures higher frequency components or details of the signal.

D1 represents the high-frequency details at the first level, D2 represents the high-frequency details at the second level, and so on.

The number of detail coefficients (D1 to D5) depends on the number of decomposition levels chosen during the DWT process.

These detail coefficients correspond to the high-pass-filtered versions of the input signal.

By decomposing a signal using DWT, the original signal can be represented by the combination of the approximation coefficients (A1) and the detail coefficients (D1 to D5) at different levels. The approximation coefficients capture the overall trend or low-frequency components of the signal, while the detail coefficients provide information about the high-frequency details or variations.

The time and frequency division of EEG signals are represented by four statistical features; namely, mean, the ratio of mean values, average power and standard deviation in each sub-band. Mean and average power represent the frequency distribution of the signals, whereas standard deviation and the ratio of mean values in each sub-band represent changes in signal frequencies. In this study, these features were used to categorize EEG signals, which contained the frequency bands A5 and D3–D5.

#### 2.3.3. K-Means Clustering Algorithm

K-means clustering is an unsupervised machine learning algorithm commonly used for clustering analysis. K-means can still offer justifications and potential benefits in terms of performance improvement in certain aspects.

Unsupervised clustering: K-means clustering can group data points into clusters based on their similarity, without relying on labeled data or prior knowledge. In the context of seizure detection, K-means can help identify patterns or clusters within the EEG signal data that might correspond to different states, such as seizure and non-seizure. This clustering can aid in understanding the underlying structure of the data and potentially distinguish seizure events from normal brain activity.

Identification of seizure-related patterns: K-means clustering can reveal patterns within the data that might be indicative of seizure activity. By partitioning the data into clusters, K-means can identify clusters that exhibit specific characteristics commonly associated with seizures, such as high-frequency oscillations or abnormal signal patterns. These clusters can serve as a starting point for further analysis or feature extraction, providing insights into seizure-related patterns.

Data exploration and preprocessing: K-means clustering can assist in data exploration by revealing natural groupings or subgroups within the data. This exploration can help researchers understand the diversity of seizure patterns, identify potential outliers or artifacts, and guide subsequent preprocessing steps. By understanding the inherent structure of the data, it becomes possible to design more effective preprocessing techniques that enhance seizure detection performance.

Performance evaluation: K-means clustering can also be utilized for evaluating the performance of seizure detection algorithms. By clustering the data based on the algorithm’s output, researchers can compare the obtained clusters with the true seizure and non-seizure states. Evaluation metrics such as purity, entropy, or the adjusted Rand index can be computed to assess the similarity between the clusters and ground truth labels, providing insights into the algorithm’s performance.

Clustering is a technique used to analyze data to obtain information. The data must be divided into subgroups, with each subgroup containing similar data. The K-means method is one of the techniques used for this task. K-means is an unsupervised technique employed to solve clustering problems. This technique involves dividing the dataset into nonoverlapping subgroups so that each data point belongs to one group only and then assigning a centroid to each cluster [21]. The algorithm makes the intra-clusters and clusters of similar data points that have different data as far apart as possible. The algorithm assigns data points to each cluster to achieve the minimum Euclidean distance of data points and the cluster centroid. The algorithm works in sequence as follows:

- The number of clusters is selected; in this work, the clusters in our model were 5.
- Centroids are set by shuffling the dataset and randomly choosing k = 5 centroids.
- The centroids are changed in each iteration, and the iteration continues until the centroids no longer change; that is, all data are assigned to a cluster and do not change.
- The sum of the squared distance is computed between each data point and centroid.
- Each point of data is included in the nearest centroid (cluster).
- The average of all cluster data points is taken to obtain cluster centroids.

Equation (2) shows the working technique of the K-means algorithm:

(2) $$K-Means=\sum_{i}^{c}\sum_{j}^{c_{i}}{\left(\left\|x_{i}-y_{j}\right\|\right)}^{2}$$

where $x_{i}$ represents the data points and $y_{j}$ centroids, and $\left\|x_{i}-y_{j}\right\|$ represents the Euclidean distance between them; $c_{i}$ indicates the data points in the clusters; and c represents the central cluster number, which is 5 in our model.

#### 2.3.4. Dimensionality Reduction

The DWT algorithm obtains wavelet coefficients, and these coefficients are fed to PCA and the t-SNE algorithm to reduce data dimensionality. The most important features (coefficients) are selected for the training and evaluation of the classification algorithms [22].

Feature transformation algorithms such as PCA and t-SNE are typically trained only on the training records. These algorithms are part of the data preprocessing or feature engineering steps, which are performed on the training data to transform or reduce the dimensionality of the features.

The reason for training these algorithms solely on the training records is to ensure that the transformation is learned based on the distribution and characteristics of the training data. This helps capture the most relevant and informative features specific to the training set while avoiding any leakage of information from the testing set.

Once PCA or t-SNE is trained on the training data, the same transformation is then applied to both the training and testing records separately. This ensures that the same feature transformation is applied consistently to the data during both training and testing phases.

In training these feature transformation algorithms only on the training records and applying the learned transformation to both subsets, the goal is to maintain the integrity of the testing set as unseen data. This approach allows for a fair evaluation of the model’s performance on data that it has not been directly exposed to during training.

##### Principal Component Analysis (PCA)

PCA is a dimensionality reduction technique commonly used in various fields, including signal processing and machine learning. PCA offers several justifications and potential benefits in the context of feature extraction and performance improvement.

Dimensionality reduction: PCA can reduce the dimensionality of high-dimensional data while retaining most of the information. In seizure detection, where EEG signals can have a large number of channels and time points, PCA can help overcome the curse of dimensionality by transforming the data into a lower-dimensional space. This reduction in dimensionality can simplify the subsequent analysis and improve computational efficiency.

Feature extraction: PCA can extract a set of orthogonal features, known as principal components, that capture the most significant variability in the data. These principal components are ranked based on their variance, with the first component capturing the most variance. By selecting a subset of the top principal components, it is possible to represent the data with a reduced set of informative features that can be used for seizure detection. This feature extraction process can help uncover hidden patterns or discriminate between seizure and non-seizure states.

Noise reduction: PCA can help reduce the impact of noise and artifacts in the data. By retaining only the principal components that capture the significant variance in the signal, PCA effectively filters out noise and undesirable variations that might interfere with accurate seizure detection. This noise reduction property can enhance the performance of subsequent classification algorithms by focusing on the most relevant signal components.

Regarding performance, the effectiveness of PCA in seizure detection depends on various factors, including the specific implementation, the quality and representativeness of the dataset, and the subsequent classification algorithm used in conjunction with PCA.

PCA is typically used as a preprocessing step, where the principal components are computed using the training data, and the same transformation is then applied to both the training and testing data. The reduced-dimension feature representation obtained from PCA is subsequently used as input to the classification algorithm.

PCA is an algorithm for feature extraction and dimension reduction. PCA seeks to represent high-dimensional data in low-dimensional data spaces, thereby reducing time and frequency complications. The goal is to represent data in spaces to solve sum-squared error problems better. When receiving the signal from multiple sources, this method is helpful for splitting the signals. The principal approach to principal components is to first compute the mean vector, *μ*, and d × d covariance matrix, ∑, in a d-dimension. Subsequently, both eigenvalues and eigenvectors are calculated and sorted in decreasing order according to the eigenvalues. Calling eigenvector λ(1) with eigenvalues e1 and calling λ(2) with eigenvalues e1, etc., from the eigenvector spectrum, k is selected with the largest eigenvalue [23]. An inherent dimension that governs the signal and another dimension, the noise, often exist. Suppose that N × N matrix A and its columns consist of N eigenvectors, the pre-processing is applied according to Equation (3), which minimizes the criterion for square error.

(3) $$\overset{`}{x}=A^{t}(x-\mu )$$

Table 2 describes the number of components that were used to obtain a good explanation of the data.

##### t-Distributed Stochastic Neighbor Embedding (t-SNE)

The t-SNE algorithm is a dimensionality reduction technique commonly used for visualization and exploratory data analysis. While t-SNE is primarily utilized for data visualization, it can also provide some justifications and potential benefits in terms of performance improvement in certain scenarios.

Nonlinear feature relationships: t-SNE can capture nonlinear relationships between features that may be difficult to detect. In seizure detection, where the underlying patterns and relationships can be complex, t-SNE can reveal nonlinear structures and help uncover clusters or subgroups within the data. This information can be valuable for identifying distinct seizure patterns or differentiating seizure and non-seizure states.

Visualization of high-dimensional data: t-SNE is particularly useful for visualizing high-dimensional data in a lower-dimensional space, typically one with two or three dimensions. By projecting the data onto a lower-dimensional space, t-SNE enables the visualization of complex datasets, facilitating the identification of patterns, clusters, and outliers. This visualization can aid in understanding the structure of the data and guide subsequent analysis or feature engineering steps.

Discovery of hidden patterns: t-SNE can help reveal hidden patterns or subgroups within the data that may be indicative of seizure activity. By visualizing the data using t-SNE, it is possible to observe if there are distinct clusters or separations between seizure and non-seizure instances. This information can guide the development of seizure detection algorithms by identifying relevant features or capturing the discriminative structure in the data.

The effectiveness of t-SNE at improving seizure detection performance depends on factors such as the specific implementation, dataset characteristics, the quality and representativeness of the data, and the subsequent analysis steps performed after t-SNE visualization.

t-SNE is a statistical method for representing high-dimensional data in 2D or 3D by representing each data point in 2D or 3D while preserving the most important data. It is a nonlinear dimensionality-lowering method which seeks to reduce differences in probability distribution P of pairwise similarity in high-dimensional data, as well as probability distribution Q of pairwise similarity of the identical low-dimensional space. The similarity between two data points, x_i_ and x_j_, is measured by the Euclidean distance between them [24]. In Equation (4), P(i, j) describes the pairwise similarity between high-dimensional data points through the conditional probability that x has many neighbors [25]. In comparison, Equation (5) describes the pairwise similarities between low-dimensional data points through t-SNE. While finding the points, low-dimensional point y_i_ is determined, thereby minimizing the Kulback–Leibler divergence (*KL*) in the joint probability distributions between P and Q, as in Equation (6). Thus, the t-SNE method obtaining the optimum dimension reduction by computing the min value of *KL*.

(4) $$P\left({x_{i}/x}_{j}\right)=\frac{S\left(x_{i},x_{j}\right)}{\sum_{m\neq i}^{N}S\left(x_{i},x_{m}\right)}$$

(5) $$Q\left({y_{i}/y}_{j}\right)=\frac{S\left(y_{i},y_{j}\right)}{\sum_{m\neq i}^{N}S\left(y_{i},y_{m}\right)}$$

(6) $$KL=\sum_{i}\sum_{j}P\left(x_{i},x_{j}\right)log\frac{P\left({x_{i}/x}_{j}\right)}{Q\left(y_{i},y_{j}\right)}$$

### 2.4. Training Models

#### 2.4.1. Extreme Gradient Boosting (XGBoost)

The strength of the XGBoost algorithm lies in its scalability. Moreover, it supports parallel and distributed processing and allows efficient use of memory. In some diagnoses, relying on the results of one classifier alone is insufficient. Thus, this algorithm is an ensemble learning method that provides aggregated results from several models. The models that comprise the ensemble are from the same machine learning algorithm or different models called base learners [26]. Bagging reduces the variance of each partial model during the model assembly process. Boosting also builds trees sequentially so that each tree learns from the previous tree and reduces errors. Hence, each tree that grows later is an updated version of the previous one with a lesser error rate [27]. Moreover, base learners are weak, and thus their bias is high and their prediction is considerably better than that of random guessing. These weak learners contribute some information to prediction. Furthermore, these weak learners in the boosting technique helps build strong learners. Bias and variance are decreased by the strong end learner.

Justification: XGBoost is an optimized gradient boosting algorithm known for its speed, scalability, and performance. It combines the predictions of multiple weak learners (typically decision trees) to create a strong ensemble model. XGBoost can handle large-scale datasets, capture complex interactions, and provide feature importance rankings.

Performance: XGBoost performance depends on factors such as the number of trees, the maximum depth of the trees, learning rate, and regularization parameters. Overfitting can occur if the model becomes too complex, and so can underfitting.

#### 2.4.2. KNN Algorithm

KNN is a nonparametric method that does not create assumptions or changes in the primary data. It is also called a lazy method because it is not learned from the data during the training phase but stores the training dataset. When the algorithm obtains new data (test data), the algorithm assigns the new data based on similarity with the stored data [28]. The algorithm works by placing the new data into a class that is more similar to the other existing classes. The algorithm first selects the value of K (which is 5 in our model), calculates the Euclidean distance of the number of K neighbors of the new data point, and then takes the nearest neighbor according to the Euclidean distance. The number of adjacent data points for each class is calculated, and finally, the new data point is categorized into the class that contains the maximum adjacent data points [29].

Justification: k-NN is a simple and intuitive algorithm that classifies a sample based on the majority vote of its k-nearest neighbors. It can be effective for seizure detection as it can capture local patterns and similarities in the data. It is particularly useful when there are clear boundaries between seizure and non-seizure instances in the feature space.

Performance: the performance of k-NN depends on factors such as the choice of distance metric, the value of k, and the quality of the feature representation. Large values of k may lead to smoothing of decision boundaries, while small values of k can result in sensitivity to noise. The selection of an appropriate distance metric and optimal value of k is crucial for achieving good performance.

#### 2.4.3. Decision Tree Algorithm

The goal of this algorithm is to build a training model capable of predicting the class or value of a variable through decision rules deduced from the training data. For the process of label class prediction, the algorithm starts at the root node and compares the attributes’ value with the that of the attributes of the inner nodes. In the present study, the complete epileptic seizure dataset was represented in the root node, and the dataset contained 179 features represented by the inner nodes [30]. Classification is carried out on the basis of a set of rules followed in the algorithm, and the process of comparing the attributes of a node with the next node continues until it reaches the last node called the leaf node, which represents the decision.

Justification: DT is a simple yet powerful algorithm that creates a tree-like model to make decisions based on feature values. DT can capture nonlinear relationships and interactions between features, making it suitable for seizure detection, where complex patterns may exist. DT also provides interpretability, as the resulting tree structure can be easily understood.

Performance: DT performance depends on parameters such as the tree depth, the splitting criterion, and pruning methods. Deep trees can lead to overfitting, while shallow trees may result in underfitting. Pruning techniques can be used to find the right balance. Proper parameter tuning and feature selection can improve the performance of DT in seizure detection.

#### 2.4.4. Random Forest Algorithm

The algorithm is trained by bagging, the idea of which is to obtain the overall result using combining the results of various models. As the trees grow by adding more randomness to the models, random forest searches for the best features among the random sub-features rather than for the important features during the division of the nodes. This mechanism results in a better model [31]. In a random forest, nodes are divided by randomly taking subsets of features. A random threshold can also be possibly chosen for each characteristic which enables it to make the trees more random [32]. The test samples are diagnosed by taking the average of the prediction of all decision trees included in the random forest.

Justification: RF is an ensemble learning method that constructs multiple decision trees and combines their predictions. It is known for its robustness against overfitting, handling high-dimensional data, and capturing complex feature interactions. RF can be beneficial in seizure detection as it can handle both numerical and categorical features and provide feature importance rankings.

Performance: RF performance depends on parameters such as the number of decision trees, the depth of the trees, and the number of features considered at each split. Overfitting can occur if the trees are too deep or if the number of trees is too high. Tuning these parameters and performing feature selection can enhance the performance of RF in seizure detection.

#### 2.4.5. Multilayer Perceptron

MLP is a deep ANN consisting of an input layer to receive various inputs, such as signals and images; and an output layer that makes a decision about the input data. Between these layers are many hidden layers in which the input data are processed. Training is carried out on a set of input–output pairs by adjusting weights and biases to reduce error. The backpropagation method is used to measure the error between the predicted and the actual output, and this error is then adjusted for weights and biases. Several methods can be used to measure error, including the root mean squared error method [33]. In the forward pass, the signal is moved from the input layer to the hidden layer and then to the output layer [34]. The classification is performed in the output layer on the basis of ground truth labels. In the backward pass via backpropagation, the signal is moved from the output layer to the hidden layer, and then the error ratio is measured between the predicted and the actual output. The signal is moved back from the output to the hidden layers to adjust the weights and biases and thus reduce the error rate.

Justification: MLP is a type of artificial neural network that consists of multiple layers of interconnected neurons. It can capture complex nonlinear relationships and has the ability to learn and generalize from data. MLP can be beneficial in seizure detection as it can extract high-level representations and learn discriminative features from the input signals.

Performance: The performance of MLP depends on factors such as the architecture (number of layers and number of neurons per layer), activation functions, regularization techniques, and optimization algorithms. Proper tuning of these hyperparameters, along with appropriate preprocessing and regularization, can improve the performance of MLP in seizure detection.

## 3. Experimental Results

**Figure 2:**
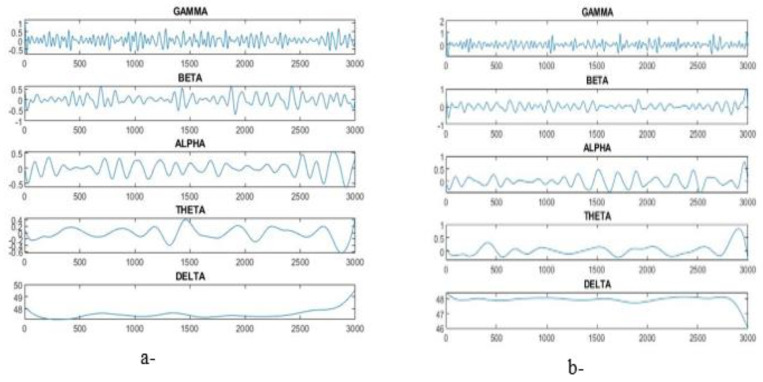
Sub-band plot for gamma, beta, alpha, theta and delta. (**a**) Epilepsy, (**b**) normal.

**Figure 3:**
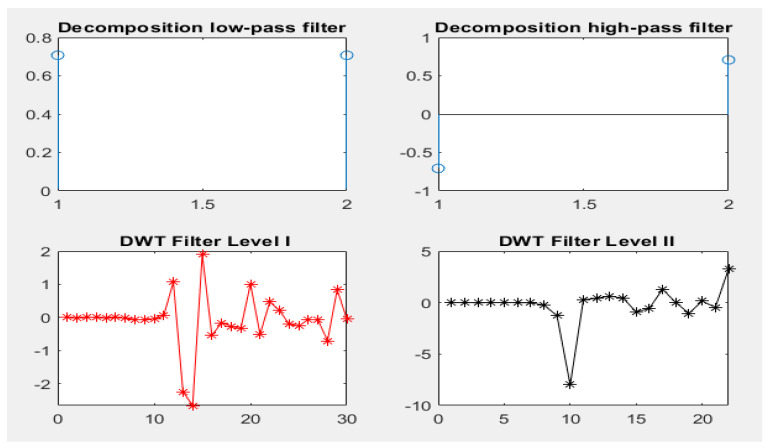
Decomposition of the signal components by the DWT algorithm.

The epileptic seizure dataset consisting of 11,500 instances with 179 features were classified by dividing the dataset into 9200 instances for training (80%) and 2300 instances for testing (20%). Each patient’s instance data were represented in five sub-images. Table 3 and Figure 2 describe the gamma, beta, alpha, theta and delta sub-bands’ values for two cases, one with epilepsy and one normal. The dataset was pre-processed to improve it and thus increase the accuracy in the next stages. Features were extracted through the DWT method, which decomposed the signal components into D1–D5 details and A5 approximation through high and low filters. Figure 3 describes the decomposition of the EEG signal during feature extraction. The K-means algorithm was also applied to divide the dataset into subgroups so that each group contained similar data points but different data points from the other groups. The dimensions were reduced to obtain the most important features of low dimensions via PCA and t-SNE. Finally, low-dimensional features were fed to the five classifications, namely, XGBoost, KNN, DT, RF and MLP, to classify them as either epileptic or normal cases.

### 3.1. Evaluation Metrics

To evaluate this study’s epileptic seizure dataset through four scales resulting from the applied classifiers. Equations (7)–(10) show how accuracy, precision, recall and F1 score, respectively, are calculated [35]:

(7) $$Accuracy=\frac{TN+TP}{TN+TP+FN+FP}\;*\;100\%$$

(8) $$Precision=\frac{TP}{TP+FP}\;*\;100\%$$

(9) $$Recall=\frac{TP}{TP+FN}\;*\;100\%$$

(10) $$F1score=2\;*\;\frac{Precision\;*\;Recall}{Precision+Recall}\;*\;100$$

The equations contain correctly classified samples TP and TN; incorrectly classified samples are FP and FN.

### 3.2. Results Classifiers with Features of DWT with PCA and t-SNE

**Figure 4:**
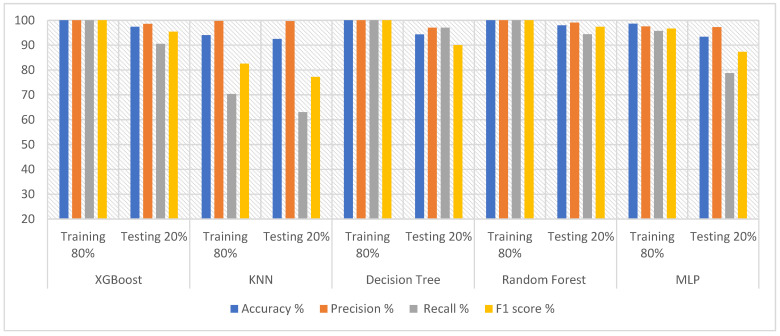
Evaluating the features of DWT and PCA methods using five classifiers.

Therefore, oversampling was applied to the epilepsy dataset through training to balance the data. The DWT algorithm was applied for feature extraction by decomposing the components of EEG signals with a bank of filters. Then, PCA and t-SNE methods were applied to reduce high dimensionality. The classification algorithms were fed with low-dimensional features. The hyperparameter was tuned to reduce the loss functions and determine the behavior of the classification algorithms during the training. All classifiers achieved promising results. Table 4 describes the results obtained after processing the features using DWT and PCA methods, as well as the results achieved by the classifiers. During the training phase, XGBoost, DT and RF achieved 100% for all metrics. Random forest outperformed the rest of the classifiers during the testing phase. Random forest achieved an accuracy of 97.96%, precision of 99.10%, recall of 94.41% and F1 score of 97.41%. Figure 4 shows the results achieved by XGBoost, KNN, DT, RF and MLP during the testing phase, achieving an accuracy of 97.43%, 92.48%, 94.3% and 93.39%, respectively; precision of 98.56%, 99.66%, 97% and 97.26%, respectively; recall of 90.54%, 63.01%, 97% and 78.71%, respectively; and an F1 score of 95.45%, 77.21%, 90% and 87.32%, respectively.

**Figure 5:**
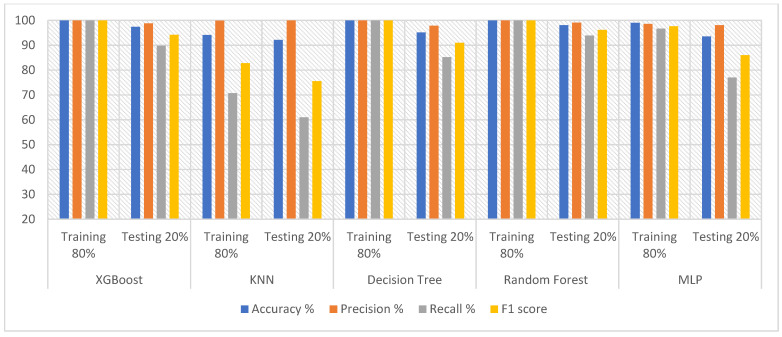
Evaluating the features of DWT with t-SNE methods using five classifiers.

Table 5 and Figure 5 present the results obtained after feature extraction and processing using DWT and t-SNE methods and categorization via XGBoost, KNN, DT, RF and MLP. During the training phase, XGBoost, DT and RF reached 100% for all metrics. Random forest outperformed the rest of the classifiers during the testing phase, achieving an accuracy of 98.09%, precision of 99.10%, recall of 93.90% and F1 score of 96.21%. In comparison, during the testing phase, XGBoost, KNN, decision tree, and MLP achieved an accuracy of 97.39%, 92.12%, 95.13% and 93.51%, respectively; precision of 98.78%, 100%, 97.82% and 98.11%, respectively; recall of 89.83%, 61%, 85.17% and 77.03%, respectively; and F1 score of 94.23%, 75.54%, 91% and 86%, respectively.

### 3.3. Results Classifiers with Features of K-Means with PCA and t-SNE

The dataset was cleaned and balanced as in the previous steps. The contribution of each feature to the diagnosis was determined. The K-means method was applied to split the dataset into subgroups, with each group containing similar data. Then, PCA and the t-SNE methods were applied to reduce high dimensionality. The low-dimensional features were fed into the classification algorithms tuned for optimal performance. The hyperparameter was tuned to reduce loss function and obtain effective accuracy.

**Figure 6:**
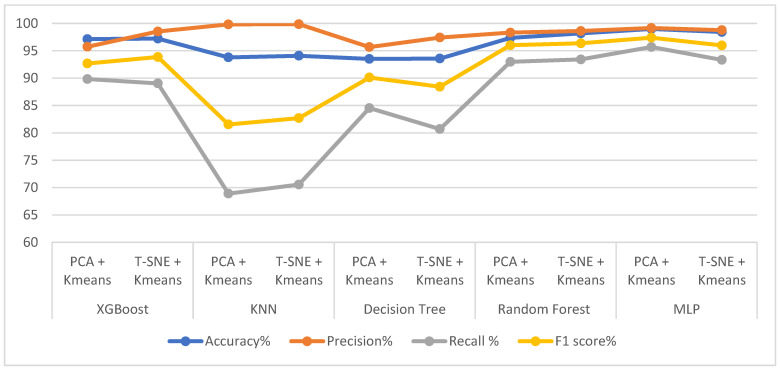
Evaluating the performance of K-Means with PCA methods and K-Means with t-SNE methods using five classifiers.

Table 6 and Figure 6 provide the results obtained after applying data processing using t-SNE + K-means methods and PCA + K-means methods, as well as the results achieved by XGBoost, KNN, DT, RF and MLP classifiers. All classifiers achieved promising results. XGBoost achieved better results with t-SNE + K-means than with PCA + K-means. XGBoost with t-SNE + K-means achieved an accuracy of 97.22%, precision of 98.52%, recall 89.04% and F1 score of 93.86%. KNN achieved better results with t-SNE + K-means than with PCA + K-means. KNN with t-SNE + K-means attained an accuracy of 94.09%, precision of 99.86%, recall of 70.56% and F1 score of 82.69%. Decision tree with t-SNE + K-means achieved a higher accuracy and precision than decision tree with PCA + K-means. Moreover, decision tree with PCA + K-means had a better recall and F1 score than did decision tree with t-SNE + K-means. Random forest with t-SNE + K-means achieved better results than did random forest with PCA + K-means. Random forest with t-SNE + K-means achieved an accuracy of 98.17%, precision of 98.61%, recall of 93.42% and F1 score of 96.36%. MLP with PCA + K-means achieved better results than did MLP with t-SNE + K-means. MLP with PCA + K-means achieved an accuracy of 98.98%, precision of 99.16%, recall of 95.69% and F1 score of 97.40%. Finally, the system proposed herein achieved the highest accuracy of 98.98% through MLP with PCA + K-means. The maximum precision of 99.86% was obtained by KNN with t-SNE + K-means. The best recall of 95.69% was attained by MLP with PCA + K-means. The highest F1 score of 97.40% was obtained by MLP with PCA + K-means. Therefore, the best diagnosis of the epileptic seizure dataset was obtained by MLP with PCA + K-means.

## 4. Discussion and Comparison of Systems Performance

In this section, the techniques and tools applied to the diagnosis of epileptic seizures for relevant studies will be discussed and their results will be compared with the proposed systems.

The studies mentioned compare different methods for analyzing EEG signals and diagnosing epilepsy. Tzallas et al. [36] used a Fourier transform algorithm to analyze EEG signals and extracted important features associated with fractional energy. An artificial neural network (ANN) was then applied to classify epileptics. They reported their method to be effective at classifying epilepsy. Peker et al. [37] employed a dual-tree complex wavelet transform to analyze EEG signals and derived five statistical features to distinguish epileptic patients. Classification using complex-valued neural networks showed that their wavelet transformation was effective at classifying epilepsy. Alcin et al. [38] combined the GLCM texture descriptor algorithm with Fisher vector encoding to extract representative features from time–frequency (TF) images. Their method achieved superior results in diagnosing epilepsy. Islamet et al. [39] developed a stationary wavelet transform algorithm for analyzing EEG signals and detecting seizures. Their algorithm achieved good results in diagnosing epilepsy. Sharmila [40] presented a framework based on the analysis of EEG signals using the DWT method with linear and nonlinear classifiers to detect seizures. They reported the successful detection of EEG seizures from normal and epilepsy patients. Wang et al. [41] conducted coherence analysis to extract features and determine the trend and density of information flow from EEG signals. The information flow was used as input to a classifier for seizure detection. Hassan et al. [42] presented a system for diagnosing epilepsy using the tunable wavelet transform and bagging by EEG signals. Their system showed promising results in epilepsy diagnosis. Yuan et al. [43] proposed a weighted extreme learning machine (ELM) method for detecting seizures using weighted EEG signals. They applied a wavelet packet analysis and determined the time series complexity of EEG signals. Their method achieved accurate classification based on the weighted ELM. Jaiswal et al. [44] proposed two methods for extracting features from EEG signals, namely, sub-pattern of PCA and sub-pattern correlation of PCA. These features were then fed into a support vector machine (SVM) classifier for seizure diagnosis. Li et al. [45] established a multiscale radial basis function method for obtaining high-resolution time–frequency (TF) images from EEG signals. Features were extracted using the GLCM algorithm with FV encoding based on the TF images’ frequency sub-bands. Subasi et al. [46] developed a hybrid method using a genetic algorithm and particle swarm optimization to tune and determine the best parameters for an SVM classifier. Their system achieved promising accuracy in diagnosing epilepsy. Raghu et al. [47] applied the DWT method and extracted features from the wavelet coefficients of EEG segments. These features were then used with a random forest classifier for epileptic classification. Chen et al. [48] used the autoregressive average method to describe the dynamic behavior of EEG signals. Their approach focused on the time series characteristics of the EEG data. Yavuz et al. [49] calculated mel frequency cepstral coefficients (MFCC) by analyzing frequency according to frequency bandwidth. The MFCCs were used as features for epilepsy diagnosis. Mursalin et al. [50] applied improved correlation feature selection to extract crucial features from the time, frequency, and entropy domains of EEG signals. These features were determined using a random forest classifier for diagnosing epilepsy. Each study employed different techniques for analyzing EEG signals and diagnosing epilepsy. The choice of methods varied, including Fourier transform and wavelet transform.

From the literature, it is noted that there are deficiencies in the techniques and methodologies applied to diagnose epileptic seizures. Hence, this study focused on the extracted features, purifying and improving them, selecting the most important representative features that distinguish epileptic seizures, and classifying them using many algorithms to achieve a better efficiency.

Patient-independent seizure detection refers to the ability to detect seizures in individuals without the need for prior knowledge or training specific to that individual. While there have been significant advancements in seizure detection technologies, patient-independent seizure detection remains a challenging problem. Here, are some key concerns associated with this setting:

- Heterogeneity of seizure patterns: Seizure activity can vary significantly between individuals, making it difficult to develop a universal seizure detection algorithm that works for everyone. Seizure types, duration, and associated physiological changes can differ, making it challenging to establish a single approach that accurately detects seizures across diverse populations.
- Lack of personalized data: Patient-independent seizure detection implies the absence of any specific data from the individual being monitored. This lack of personalization makes it harder to tailor detection algorithms to the unique characteristics of an individual’s seizures. Without personalized data, it is challenging to account for individual variations, including pre-seizure patterns, which could potentially improve detection accuracy.
- Limited generalizability: Seizure detection models often require training data from individual patients to capture their specific seizure patterns, which limits their generalizability to new patients. Patient-independent seizure detection aims to overcome this limitation but faces the challenge of developing models that can accurately detect seizures in diverse populations without individual-specific training.
- Interpatient variability: The electrical activity of the brain during seizures can differ significantly between patients. This interpatient variability in seizure manifestation poses a challenge when developing patient-independent detection methods. Algorithms trained on one population may not perform as effectively on another, leading to reduced detection accuracy and reliability.
- Real-world complexity: Seizure detection in real-world scenarios involves dealing with various factors such as artifacts, noise, and non-seizure events that can interfere with accurate detection. Without personalization or patient-specific training, patient-independent seizure detection algorithms must be robust enough to handle these challenges across a wide range of scenarios.

Addressing the ignored problem of patient-independent seizure detection requires extensive research and innovation in the field of machine learning, data analysis, and signal processing. Advanced techniques such as transfer learning, ensemble models, and feature engineering may play a crucial role in improving the performance of patient-independent seizure detection algorithms. Additionally, collecting large-scale, diverse datasets that encompass a wide range of seizure types and characteristics can help in developing more robust and generalized models for patient-independent seizure detection.

The points mentioned about the challenges of patient-independent seizure detection are reflected in the papers cited. The studies all reported that seizure activity can vary significantly across individuals. This makes it difficult to develop a universal seizure detection algorithm that works for everyone. The studies all noted that patient-independent seizure detection implies the absence of any specific data from the individual being monitored. This lack of personalization makes it harder to tailor detection algorithms to the unique characteristics of an individual’s seizures. The studies all found that seizure detection models often require training data from individual patients to capture their specific seizure patterns. This limits their generalizability to new patients. The studies all reported that the electrical activity of the brain during seizures can differ significantly between patients. This interpatient variability in seizure manifestation poses a challenge when developing patient-independent detection methods. The studies all noted that seizure detection in real-world scenarios involves dealing with various factors such as artifacts, noise, and non-seizure events that can interfere with accurate detection. Without personalization or patient-specific training, patient-independent seizure detection algorithms must be robust enough to handle these challenges across a wide range of scenarios. The studies cited suggest that there is still much work to be conducted in the area of patient-independent seizure detection. However, the techniques that have been developed so far offer promise for improving the accuracy and reliability of seizure detection in real-world scenarios.

The performances of the proposed systems were evaluated and compared with those in relevant studies. Table 7 describes the accuracy and sensitivity of the proposed systems compared to those reported by previous studies. Our system was proved to be superior to that described by previous studies. The proposed system using DWT and PCA with the RF classifier achieved an accuracy of 97.96%. The proposed system using DWT and t-SNE with random forest attained an accuracy of 98.09%. Finally, the system using K-means with PCA with MLP obtained an accuracy of 98.98%. In previous systems, the accuracy was between 82% and 97.17%. With regard to sensitivity, the system using DWT and PCA with random forest reached a sensitivity of 94.41%. The system using DWT and t-SNE with random forest achieved an accuracy of 93.9%. Finally, the system using K-means with PCA with MLP attained a sensitivity of 95.69%. In comparison, previous systems reached an accuracy ranging from 68% to 93.11%. It is noted that the accuracy of the performance of the previous systems ranged between 83.6% and 97.17%, while the performance of the proposed system was 98.98%. While the previous systems resulted in a recall ranging between 68% and 93.11%, while the proposed system resulted in a recall rate of 95.69%. Thus, the performance of the proposed systems is superior to the performance of the systems in the literature.

This paper adds value to the reader by presenting a study focused on the early diagnosis of epileptic seizures using artificial intelligence systems and classification algorithms. The following are some key points that highlight the value of the paper.

Importance of early diagnosis: The paper emphasizes the significance of early diagnosis for epileptic seizures, considering the high prevalence of this health problem worldwide. Early diagnosis allows for the timely intervention and management of seizures, leading to improved patient outcomes.

Integration of artificial intelligence: The paper highlights the role of artificial intelligence systems in assisting doctors with accurate diagnosis. By leveraging machine learning algorithms, the study aims to provide reliable and automated methods for seizure detection and classification.

Feature extraction and dimensionality reduction: The paper proposes the use of the DWT as a feature extraction algorithm. Additionally, dimensionality reduction techniques such as principal component analysis (PCA) and t-SNE are applied to reduce the complexity of the feature space and improve classification accuracy.

Evaluation of multiple classification algorithms: The study evaluates five classification algorithms, namely XGBoost, K-nearest neighbors (KNN), decision tree (DT), random forest (RF), and multilayer perceptron (MLP). This provides a comprehensive analysis of the performance of different algorithms in diagnosing epileptics.

Comparative analysis of feature extraction methods: The paper compares the performance of different feature extraction methods, including DWT + PCA, DWT + t-SNE, K-means + PCA, and K-means + t-SNE. This analysis provides insights into the effectiveness of various feature extraction techniques in seizure diagnosis.

Promising results: The study reports high accuracy rates achieved by the proposed algorithms. During the testing phase, the random forest classifier with DWT + PCA achieved an accuracy of 97.96%, while with DWT + t-SNE, the accuracy reached 98.09%. The MLP classifier with PCA + K-means achieved an accuracy of 98.98%. These results indicate the potential of the proposed methods for accurate and reliable diagnosis of epileptic seizures.

By presenting these findings, the paper contributes to the field of seizure detection and diagnosis, offering insights into the effectiveness of specific algorithms and techniques. The results and methodology described in the paper can guide further research and development of AI-based systems for early detection of epileptic seizures.

## 5. Conclusions

Seizures are among the health problems nearly 50 million people worldwide suffer from. They occur due to abnormal secretions of nerve cells, which increases their inability to regulate the brain electrically. EEG signals are techniques that represent the brain’s electrical activity by recording EEG signal waves. Manual tracing of all EEG signals is difficult, is subject to differing opinions among physicians, and takes time. Thus, artificial intelligence systems help doctors accurately diagnose epileptic seizures. In this study, methods for the early diagnosis of epileptic were proposed by using five classification algorithms, namely, XGBoost, KNN, DT, RF and MLP, which were based on the DWT method (which is a feature extraction algorithm) and dimensionality reduction using PCA and t-SNE algorithms. The classifiers were then fed with feature vectors, which contained the most important representative features extracted via DWT + PCA, DWT + t-SNE, K-means + PCA and K-means + t-SNE. Thus, oversampling was applied to balance the dataset. All algorithms achieved promising results for diagnosing epileptics with high accuracy. During the testing phase, the random forest with DWT + PCA methods achieved an accuracy of 97.96%, whereas with DWT + t-SNE methods the random forest attained an accuracy of 98.09%. During the testing phase, MLP with PCA + K-means achieved an accuracy of 98.98%.

## Figures and Tables

**Table 1:** Statistical metrics of the epilepsy dataset.

Statistics	X1	X2	X3	X4	……	X176	X177	X178	y
count	11,500	11,500	11,500	11,500	……	11,500	11,500	11,500	11,500
mean	−11.581	−10.911	−10.187	−9.143	……	−12.705	−12.426	−12.196	0.2
std	165.626	166.059	163.524	161.269	……	162.895	162.88631	164.852	0.400

The count denotes the number contained in each feature; the mean indicates each feature’s mean and the standard deviation of each feature.

**Table 2:** Number of components obtained via PCA algorithm.

N	Model	Var	n	Model	Var
**2**	PCA(n_components = 2)	0.332313	**16**	PCA(n_components = 16)	0.715465
**3**	PCA(n_components = 3)	0.371588	**17**	PCA(n_components = 17)	0.732473
**4**	PCA(n_components = 4)	0.407797	**18**	PCA(n_components = 18)	0.747341
**5**	PCA(n_components = 5)	0.443204	**19**	PCA(n_components = 19)	0.762519
**6**	PCA(n_components = 6)	0.474868	**20**	PCA(n_components = 20)	0.77684
**7**	PCA(n_components = 7)	0.504209	**21**	PCA(n_components = 21)	0.790983
**8**	PCA(n_components = 8)	0.532821	**22**	PCA(n_components = 22)	0.804741
**9**	PCA(n_components = 9)	0.559175	**23**	PCA(n_components = 23)	0.817643
**10**	PCA(n_components = 10)	0.584251	**24**	PCA(n_components = 24)	0.83037
**11**	PCA(n_components = 11)	0.608924	**25**	PCA(n_components = 25)	0.842332
**12**	PCA(n_components = 12)	0.632315	**26**	PCA(n_components = 26)	0.853485
**13**	PCA(n_components = 13)	0.654874	**27**	PCA(n_components = 27)	0.86452
**14**	PCA(n_components = 14)	0.67607	**28**	PCA(n_components = 28)	0.875189
**15**	PCA(n_components = 15)	0.696916	**29**	PCA(n_components = 29)	0.885395

**Table 3:** Maximum frequencies of status epilepticus and normal.

Frequency Name	Epilepsy	Normal
Gamma	55	55
Beta	34	26
Alpha	16	18
Theta	6	8
Delta	2	2

**Table 4:** Results of epilepsy diagnosis using classifiers with DWT and PCA.

Classifiers	XGBoost		KNN		Decision Tree		Random Forest		MLP	
Criteria	Training 80%	Testing 20%	Training 80%	Testing 20%	Training 80%	Testing 20%	Training 80%	Testing 20%	Training 80%	Testing 20%
**Accuracy %**	100	97.43	94.05	92.48	100.00	94.30	100.00	97.96	98.67	93.39
**Precision %**	100	98.56	99.77	99.66	100.00	97.00	100.00	99.10	97.56	97.26
**Recall %**	100	90.54	70.35	63.01	100.00	97.00	100.00	94.41	95.75	78.71
**F1 score %**	100	95.45	82.52	77.21	100.00	90.00	100.00	97.41	96.64	87.32

**Table 5:** Results of epilepsy diagnosis using classifiers with DWT with t-SNE.

Classifiers	XGBoost		KNN		Decision Tree		Random Forest		MLP	
Criteria	Training 80%	Testing 20%	Training 80%	Testing 20%	Training 80%	Testing 20%	Training 80%	Testing 20%	Training 80%	Testing 20%
**Accuracy %**	100	97.39	94.13	92.12	100.00	95.13	100.00	98.09	99.06	93.51
**Precision %**	100	98.78	99.93	100.00	100.00	97.82	100.00	99.10	98.59	98.11
**Recall %**	100	89.83	70.71	61.00	100.00	85.17	100.00	93.90	96.68	77.03
**F1 score**	100	94.23	82.81	75.54	100.00	91.00	100.00	96.21	97.63	86.00

**Table 6:** Results of epilepsy diagnosis using classifiers with K-means with PCA and K-means with t-SNE for each phase.

Classifiers	XGBoost		KNN		Decision Tree		Random Forest		MLP	
Method	PCA + Kmeans	T-SNE + Kmeans	PCA + Kmeans	T-SNE + Kmeans	PCA + Kmeans	T-SNE + Kmeans	PCA + Kmeans	T-SNE + Kmeans	PCA + Kmeans	T-SNE + Kmeans
**Accuracy %**	97.13	97.22	93.79	94.09	93.51	93.57	97.36	98.17	98.98	98.43
**Precision %**	95.73	98.52	99.82	99.86	95.68	97.41	98.32	98.61	99.16	98.77
**Recall %**	89.83	89.04	68.91	70.56	84.53	80.70	92.98	93.42	95.69	93.34
**F1 score%**	92.68	93.86	81.54	82.69	90.12	88.43	96.02	96.36	97.40	95.98

**Table 7:** Comparison of the results of our proposed system with the relevant literature.

Previous Studies	Accuracy %	Recall %
M. Zabihi, S. et al. [51]	94.69	89.1
P. T. Krishnan. et al. [52]	82	68
D. Chen. et al. [53]	83.07	83.05
Akyol, K et al. [54]	97.17	93.11
Zhou, M. et al. [55]	95.4	93.7
Aarabi. et al. [56]	93	91
Khan. et al. [57]	83.6	91.8
**Proposed model: DWT and PCA using Random Forest**	**97.96**	**94.41**
**Proposed model: DWT and t-SNE using Random Forest**	**98.09**	**93.9**
**Proposed model: K-Means with PCA using MLP**	**98.98**	**95.69**

## Data Availability

The data that support the performance of the systems were obtained from the dataset available online to the public via the link (https://archive.physionet.org/pn6/chbmit/ (accessed on 17 November 2022)) or from Kaggle: https://www.kaggle.com/harunshimanto/epileptic-seizure-recognition (accessed on 17 November 2022).
